# Comparative Analysis of the Immunogenicity and Protective Effects of Inactivated EV71 Vaccines in Mice

**DOI:** 10.1371/journal.pone.0046043

**Published:** 2012-09-28

**Authors:** Qunying Mao, Chenghong Dong, Xiuling Li, Qiang Gao, Zengbing Guo, Xin Yao, Yiping Wang, Fan Gao, Fengxiang Li, Miao Xu, Weidong Yin, Qihan Li, Xinliang Shen, Zhenglun Liang, Junzhi Wang

**Affiliations:** 1 National Institutes for Food and Drug Control, Beijing, China; 2 Institute of Medical Biology, Chinese Academy of Medical Sciences, Kunming, China; 3 National Vaccine and Serum Institute, Beijing, China; 4 Sinovac Biotech Co., Ltd., Beijing, China; 5 Hualan Biological Engineering Inc, Henan, China; University of Illinois at Chicago, United States of America

## Abstract

**Background:**

Enterovirus 71 (EV71) is the major causative agent of hand, foot, and mouth disease (HFMD). Three inactivated EV71 whole-virus vaccines of different strains developed by different manufacturers in mainland China have recently entered clinical trials. Although several studies on these vaccines have been published, a study directly comparing the immunogenicity and protective effects among them has not been carried out, which makes evaluating their relative effectiveness difficult. Thus, properly comparing newly developed vaccines has become a priority, especially in China.

**Methods and Findings:**

This comparative immunogenicity study was carried out on vaccine strains (both live and inactivated), final container products (FCPs) without adjuvant, and corresponding FCPs containing adjuvant (FCP-As) produced by three manufacturers. These vaccines were evaluated by neutralizing antibody (NAb) responses induced by the same or different dosages at one or multiple time points post-immunization. The protective efficacy of the three vaccines was also determined in one-day-old ICR mice born to immunized female mice. Survival rates were observed in these suckling mice after challenge with 20 LD_50_ of EV71/048M3C2. Three FCP-As, in a dose of 200 U, generated nearly 100% NAb positivity rates and similar geometric mean titers (GMTs), especially at 14–21 days post-inoculation. However, the dynamic NAb responses were different among three vaccine strains or three FCPs. The FCP-As at the lowest dose used in clinical trials (162 U) showed good protective effects in suckling mice against lethal challenge (90–100% survival), while the ED_50_ of NAb responses and protective effects varied among three FCP-As.

**Conclusions:**

These studies establish a standard method for measuring the immunogenicity of EV71 vaccines in mice. The data generated from our mouse model study indicated a clear dose-response relationship, which is important for vaccine quality control and assessment, especially for predicting protective efficacy in humans when combined with future clinical trial results.

## Introduction

Enterovirus 71 (EV71) is a small RNA virus belonging to the *Enterovirus* genus. It is a spherical particle with icosahedral (cubic) symmetry and contains a positive-sense single-stranded RNA approximately 7.4 kb long. Each subunit of the viral capsid contains a copy of the four structural viral proteins (VP1–VP4); VP1, VP2, and VP3 are external, while VP4 is completely within the interior of the viral particle and is not, therefore, exposed to the host antibody response [1]. VP1 displays the predominant neutralizing epitope [2]. EV71 infection mainly leads to hand, foot, and mouth disease (HFMD) and EV71-associated neurological diseases, including aseptic meningitis, brainstem encephalitis, and acute flaccid paralysis indistinguishable from poliomyelitis [3]. In recent years EV71 has caused epidemics and is a growing public health concern due to a high incidence of severe symptoms and high fatality rates in Asia-Pacific regions [4]–[13]. Because of the lack of preventative and therapeutic measures, the development of safe and effective EV71 vaccines has become an urgent matter, especially in China. Currently, there are several commercial manufacturers and research institutes developing different types of EV71 vaccines, including inactivated virus vaccines, attenuated live vaccines, engineered virus-like particle (VLP) vaccines, and polypeptide vaccines [14]–[21]. Benefiting from the research community's extensive experience in developing other enterovirus vaccines, such as the polio and hepatitis A vaccines, development of inactivated virus vaccines has proceeded faster than the others and exhibits the highest apparent immunogenicity [18], [20], [22]. In mainland China [23], Taiwan [24] and Singapore [25], these inactivated virus vaccines have been tested in clinical trials and are expected to be the first class of vaccines to be employed to prevent EV71-associated diseases worldwide [23].

In mainland China, three inactivated EV71 vaccines have been developed by different manufacturers. Although the three vaccines are all inactivated virus vaccines, differences in their manufacturing processes exist, including the strains (though all three are the C4 genotype), cell substrate (Vero or diploid cells), cell culture system (roller bottles, cell factories or microcarrier bioreactor system), production process, and vaccine dose (Table 1). All these factors may lead to differences in immunogenicity [15], [26]. Although good immunogenicity and protective effects have been reported at particular time points after immunization, the antigen content of these vaccines was reported in different units (µg/ml, KU/ml, EU/ml), and different animal models were empolyed by the different manufacturers to test these vaccines [27], [28]. These differences make it difficult to compare the immunogenicity and protective effects among the different EV71 vaccines, which will be important for testing in clinical trials. A prior collaborative effort was carried out to standardize the EV71 antigen content of three aqueous bulk and three final container products (FCPs) without adjuvant from three manufacturers (unpublished data). Based on the standardized results of the collaborative study, experiments were carried out to compare the immunogenicity and protective effects of EV71 vaccine antigens from the three different manufacturers at different production stages, including the vaccine strains themselves, FCP, and FCP with alum adjuvant (FCP-A). Additionally, the relationship between NAb response and protective effect was determined. These studies provide a basis for the design of clinical trials to confirm dosage and evaluate the protective effects of EV71 vaccines.

**Table 1:** Properties of EV71 vaccines developed by three different manufacturers.

Vaccine manufacturer	Vaccine strain*				Cell substrate	Production process	Protective agent in FCP	Adjuvant and protective agent in FCP-A
	Genotype	Gene mutation rate (VP1/VP2/VP3)	Amino acid mutation rate (VP1/VP2/VP3)	Virus titer (lg PFU/ml)				
A	C4	96.3/96.1/95.9	97.6/99.2/99.6	7.19	KMB_17_	Spinner bottle	Glycine	Aluminium hydroxide, Glycine
B	C4	96.6/95.8/97.0	97.6/99.6/99.6	6.98	Vero	Microcarrier bioreactor system Fermentation cylinder	Human immunoglobulin	Aluminium hydroxide
C	C4	96.4/96.5/96.3	97.6/99.2/100	6.58	Vero	Cell factory	None	Aluminium hydroxide

* Ribonucleotide sequence homologies between the vaccine strains and a reference strain BJ08 were determined (GenBank accession no: FJ828519).

## Materials and Methods

### 1. Collaborative laboratories

The following laboratories were involved in this collaborative study comparing the EV71 vaccines: the National Institutes for Food and Drug Control of China (lab 1), the National Vaccine & Serum Institute (lab 2), and Sinovac Biotech Co., Ltd., Beijing (lab 3).

### 2. Testing for antigen content

The EV71 antigen content of three aqueous bulks and three FCPs from three manufacturers (A, B, C; Table 1) was assayed in a previous collaborative study in the four labs (the same three labs in this study and the lab of the Institute of Medical Biology, Chinese Academy of Medical Sciences). A quantitative ELISA assay kit [29] was used to detect the EV71 antigen content using the reference standard (1600 U/ml) provided by the National Institutes for Food and Drug Control [30]. The samples and EV71 antigen standard (1600 U/ml) were serially diluted two-fold and tested in duplicate wells. Variance analysis (*F*-test) was performed to determine the linearity and parallelism of the samples and the EV71 antigen standard. Only when both *P* values were greater than 0.05, were the EV71 antigen reference and the samples considered to have a parallel linear relationship. The parallel-line method was used to calculate the antigen content of the samples. Results are expressed in standard national EV71 antigen units/ml (U/ml).

### 3. EV71 vaccine strains

The three vaccine strains (labeled M1, M2, and M3: C4 genotype) came from three different vaccine manufacturers in mainland China herein termed A, B and C (because the vaccines are still in clinical trials, we have kept the manufacturers' names anonymous). M1, M2, and M3 were isolated from EV71 viruses in HFMD epidemic areas in mainland China since 2008 (Table 1). The sequence homologies between the VP1 region of the three vaccine strains (M1, M2, and M3) and the reference strain BJ08 (GenBank accession no: FJ828519) were 97.4%, 96.6%, and 97.9%, respectively [26]. The virus titers of M1, M2, and M3 were 7.19, 6.98 and 6.58 lg PFU/ml [26], and the antigen concentrations were 626, 127, and 332 U/ml (unpublished data), respectively.

The three inactivated EV71 strains (labeled IM1, IM2, and IM3) were treated with formalin (0.25% wt/vol) at 37°C for 3 d. All EV71 strains and inactivated EV71 strains were stored at −80°C before use. Appropriate amounts of Minimum Essential Media (MEM) were used to dilute each to an equivalent EV71 virus titer of 6.50 lg PFU/ml. Samples were blinded and distributed by lab 1 to the collaborative labs for mouse immunization and testing of the NAb response.

### 4. EV71 FCPs

Three EV71 aqueous bulks (Q1, Q2 and Q3) were produced by manufacturers A, B, and C and derived from M1, M2, and M3 vaccine strains, respectively, using their own processing techniques (Table 1). The purity of all three aqueous bulks was verified by HPLC to be above 95%. Three EV71 FCPs (B1, B2, and B3) were diluted by the manufacturers (A, B, and C) from aqueous bulks in PBS containing the protective agent (Table 1). The EV71 antigen content of the three EV71 FCPs and three aqueous bulks was assayed three consecutive times in a previous collaborative study using the quantitative ELISA kit [29] mentioned above with the EV71 antigen reference standard (1600 U/ml, from the National Institutes for Food and Drug Control) [26]. The EV71 antigen content of the three FCPs ranged from 506 to 1047 U/ml (CV: 4.8%–12.7% by collaborative labs), as measured by the parallel-line method. EV71 FCPs were stored at 4°C before use. Appropriate amounts of PBS were used to dilute each to an equivalent EV71 antigen content of 500 U/ml. Samples were blinded and distributed by lab 1 to the collaborative labs.

### 5. EV71 FCP-As

Three FCP-As (V1, V2, and V3) were produced through the absorption of aqueous bulks onto alum adjuvant by manufacturers A, B, and C (Table 1). The EV71 antigen content of the three FCP-As varied between 324 and 1012 U/ml (162–506 U/dose/0.5 ml) which was calculated according to the previous collaborative study on EV71 antigen content of the three aqueous bulks. EV71 FCP-As were stored at 4°C before use. Appropriate amounts of alum adjuvant were used to dilute each vaccine to an equivalent EV71 antigen concentration of 200 U/ml (for comparative studies of NAbs responses) or 324 U/ml (for protection studies). Samples were blinded and distributed by lab 1 to the collaborative labs.

### 6. Comparative studies of NAbs induced in mice by EV71 viral strains, FCPs, and FCP-As from three different manufacturers

**Figure 1:**
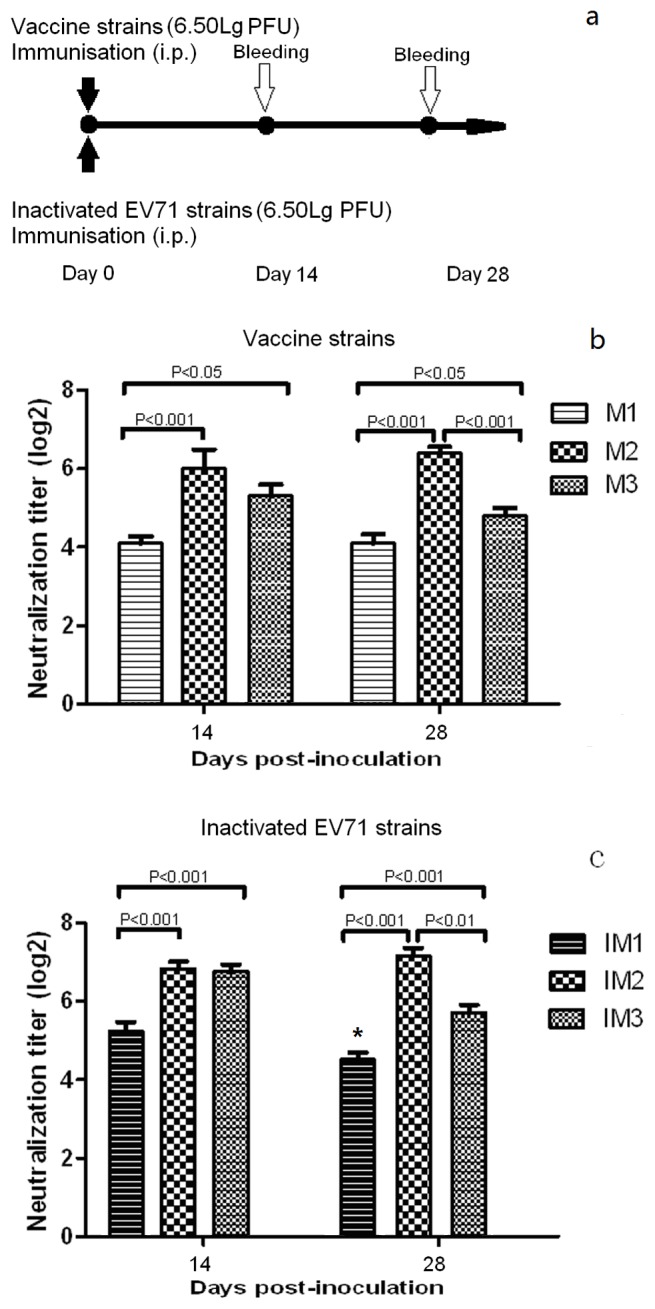
Dynamic trend of neutralizing antibody GMTs for EV71 strains. Female BALB/c mice (4–6 weeks old) were i.p. inoculated with three vaccine strains (M1, M2, and M3) and three inactivated EV71 strains (IM1, IM2, and IM3) containing 6.50 lg CCID_50_ per dose. NAb were detected at 14 d and 28 d after inoculation. NAb titers equal to and above 1∶1536 were assigned a value of 1∶1536. Common logarithmic transformation of the NAb titer raw data was used to calculate the GMT and CV. SPSS_10.0_ software was used for statistical analyses. * With the exception of the NAb induced by IM1 at 28 d, NAbs induced by inactivated EV71 were significantly higher than those of live EV71 (*P<0.05*).

The mice were immunized with the blinded samples at the three labs according to the same protocol. Female BALB/c mice (4–6 weeks old) were obtained from Vital River Laboratories, Ltd. Beijing, China. Samples of the three vaccine strains (M1, M2, and M3), inactivated EV71 strains (IM1, IM2, and IM3), FCPs (B1, B2, and B3) and EV71 FCP-As (V1, V2, and V3) containing equivalent virus titer or antigen content in a volume of 1.0 ml were used to immunize mice intraperitoneally (i.p.). Each group receiving EV71 vaccine strains or inactivated EV71 strains contained 20 mice; the sera were collected 14 d and 28 d post-inoculation (10 mice/time point). The groups receiving FCPs each included 60 mice, and the sera were collected at six time points: 7, 14, 21, 28, 56 and 84 d post-inoculation (10 mice/time point). The sera were isolated and tested by each lab with the identical standard operating procedure (SOP). In all experiments, the corresponding diluent was used as a negative control (Figure 1a, Figure S1 and Table S1).

### 7. ED_50_ of three FCP-As at 7 d after inoculation

The blinded samples were used to immunize mice according to the same protocol in the three labs. Female BALB/c mice (4–6 weeks old) were obtained from Vital River Laboratories, Ltd. Beijing, China. The alum adjuvant was used to serially dilute the three FCP-As (V1, V2, and V3) four times from 200 U/ml to 0.8 U/ml. Each diluted FCP-A was used to i.p. immunize mice with an injection volume of 1.0 ml. Each group contained 10 mice, from which the sera were collected at 7 d after inoculation. The sera were isolated and tested by each lab with the same SOP. In all experiments, the corresponding diluent was used as a negative control.

### 8. EV71 NAb assay

The titer of NAb against EV71 was measured in all samples with the cytopathogenic effect (CPE) assay performed with the same SOP at each lab [31], [32]. The protocol utilized in this experiment was a modified version of the one used for polioviruses (WHO 1997) [33]. Briefly, blood samples were diluted 1∶8, and the serum was inactivated at 56±0.5°C for 30 min. Fifty microliters of each serum dilution (ranging from 1∶8 to 1∶1024) was mixed with 100 TCID_50_ EV71 (EV71/523-07T, C4 genotype) per well in a 96-well microplate (Thermo Fisher Scientific, NUNC, Denmark) and incubated at 37±0.5°C for 2 h. Next, a 100 µl suspension of rhabdomyosarcoma cells (RD cells: ATCC, CCL-136, a gift from the National Vaccine & Serum Institute) (1×10^5^ cells/ml) was added per well. Each assay set a cell control, a virus control (no serum) and EV71 NAb standards (one quantitative standard and three reference sera). The plates were placed in a CO_2_ incubator at 35±0.5°C for 7 days after which CPEs were observed by microscopy [26]. NAb titers were defined as the highest dilution capable of inhibiting 50% of the CPEs. Only the results of assays in which each control fit the specifications were considered valid. NAb titers against EV71 were defined as positive if equal to or greater than 1∶8. NAb titers equal to and above 1∶1536 were assigned a value of 1∶1536. All samples were assayed in a blinded manner and were double-checked by a second investigator. The results of experiments from all labs were collected and analyzed by lab 1.

### 9. Evaluation of protective efficacy in suckling mice

**Figure 2:**
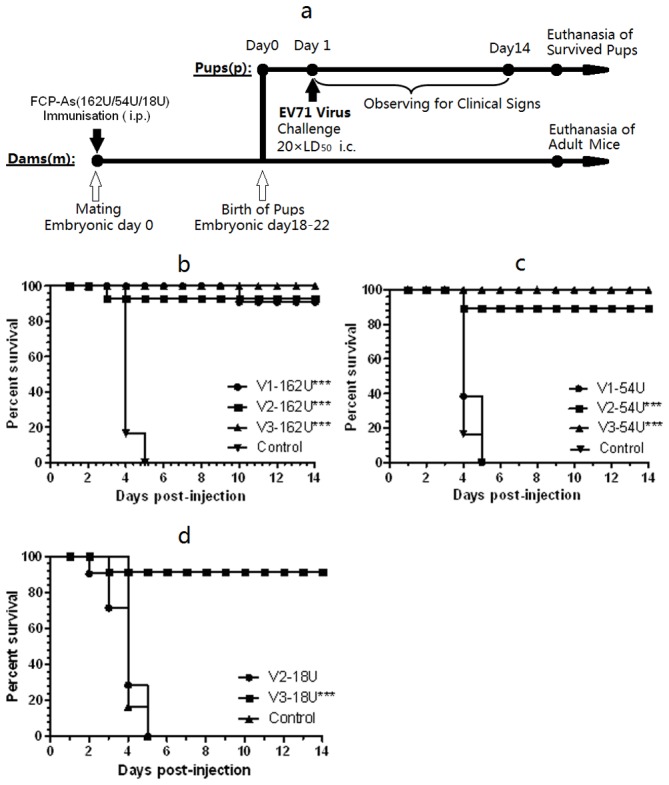
Protection of EV71 inoculated suckling mice by three vaccines administered to dams. Female ICR mice (9–10 weeks old) were divided into three groups and immunized with V1, V2 or V3 at doses of 162 U/0.5 ml/mouse, 54 U/0.5 ml/mouse, and 18 U/0.5 ml/mouse by i.p. injection. Aluminum salt adjuvant and inactivated CA16 virus solution (G-10, titer: 10^7.5^ TCID_50_/ml) were used as negative and CA16 controls, respectively. After immunization of the female mice with vaccines V1, V2, or V3 at the dose of 162 U/0.5 ml/mouse (**b**), 54 U/0.5 ml/mouse (**c**), or 18 U/0.5 ml/mouse (**d**), they were caged and mated with naïve males. On the first postnatal day, EV71/048M3C2 was administered to the ICR suckling mice intracerebrally at 20 LD_50_. After infection, the suckling mice were observed daily to determine the protective effect of maternal antibody transfer against EV71 challenge, and the survival rates were calculated. Data shown are representative of three independent experiments.

Female ICR mice (9–10 weeks old) were obtained from Vital River Laboratories, Ltd. Beijing, China. As shown in Figure 2a, for each vaccine (V1, V2, or V3) female mice were divided into three groups and immunized at three different doses (162 U/0.5 ml/mouse, 54 U/0.5 ml/mouse, and 18 U/0.5 ml/mouse) by i.p. injection. Aluminum salt adjuvant and inactivated CA16 virus solution (G-10, titer: 10^7.5^ TCID_50_/ml) were used as a negative control and a CA16 control, respectively. One hour after immunization, the female mice were caged and mated with naïve males. Pregnant dams delivered pups 21–28 d post first immunization. On the first postnatal day, EV71/048M3C2 (mouse-adapted strain, C4 genotype, provided by the National Vaccine & Serum Institute) was administered intracerebrally to all newborn suckling mice at 20 times the median lethal dose (LD_50_). The suckling mice were then observed for 14 days, recording their health, disease onset, and death rate. The median effective dose (ED_50_) was calculated for each experimental group based on the survival rates of the newborn suckling mice. Results were only considered valid if the death rate in the negative control group reached 90% within 14 days. Two independent experiments were performed; due to good repeatability, the results were combined for statistical analysis.

### 10. Statistical methods

EV71 antigen content was measured and calculated using the parallel-line method with the EV71 antigen reference standard (1600 U/ml). Variance analysis (*F*-test) was performed to determine the linearity and parallelism of the sample and standard EV71 antigen. Only when both *P* values were greater than 0.05, were the antigen reference and the sample considered to have a parallel linear relationship. Results are expressed in standard national EV71 antigen units/ml (U/ml) [30]. ED_50_ was calculated using the Spearman-Karber method [34]. A comparison of EV71 NAb positive rates was performed with the χ^2^ test. Common logarithmic transformation of the NAb titer raw data was used to calculate the geometric mean titer (GMT) and coefficient of variance (CV). SPSS_10.0_ software was used for statistical analyses. Data were considered significant at *P*<0.05. The results were highly repeatable in the three labs and were combined for statistical analysis.

## Results

### 1. NAb responses to EV71 vaccine strains

As shown in Table 2, the NAb seroconversion rates of mice inoculated with the three EV71 vaccine strains and inactivated EV71 (6.50 lg PFU/ml/mouse) ranged from 90.0% to 100.0% at 14 and 28 d after immunization. However, the NAb GMTs were significantly different at each time point with the same order: M2>M3>M1 and IM2>IM3>IM1 (Figure 1b and 1c). The anti-EV71 NAb GMTs elicited by M1, M2, and M3 were 1∶14.9, 1∶54.5, and 1∶33.2, respectively, at 14 d post-inoculation. The differences in GMTs among the three groups varied between 1.6- to 3.7-fold. The respective Nab GMTs were 1∶15.3, 1∶67.1, and 1∶23.4 (1.5- to 4.4-fold difference among the three virus strains) at 28 d after inoculation, and no sharp decreases were observed from 14 to 28 d.

**Table 2:** NAb seropositivity rate (dilution ≥1∶8) of mice inoculated with live and inactivated EV71 vaccine strains from three difference manufacturers.

Sample	Manufacturer (Code)	Seropositive rate (%)	
		14 d	28 d
EV71 strains (6.50 lg PFU/mouse)	A(M1)	100.0 (10/10)	100.0 (10/10)
	B(M2)	90.0 (9/10)	100.0 (10/10)
	C(M3)	100.0 (10/10)	100.0 (10/10)
Inactivated EV71 strains (6.50 lg PFU/mouse)	A(IM1)	96.7 (29/30)	90.0 (27/30)
	B(IM2)	100.0 (30/30)	100.0 (30/30)
	C(IM3)	100.0 (30/30)	96.7 (29/30)

Immunization and NAb detection were carried out in three collaborative labs using the same SOP. Ten to thirty mice were immunized in each dose group. The results were highly repeatable between the three labs and were combined for statistical analysis.

For the inactivated EV71 strains, the NAb GMTs induced by IM1, IM2, and IM3 were 1∶38.4, 1∶114.1, and 1∶107.9 (a 1.0- to 3.0-fold difference) and 1∶23.0, 1∶143.7, and 1∶52.6 (a 2.3- to 6.2-fold difference) at 14 d and 28 d post-inoculation, respectively. With the exception of NAbs induced by M1 at 28 d, NAbs induced by inactivated EV71 at both 14 and 28 d post-inoculation were higher than that of live EV71 (*P*<0.05), indicating that formalin inactivation increased the immunogenicity of EV71.

Comparing the immune response to the three EV71 strains, both live and inactive, the lowest NAb GMTs were observed with the M1 and IM1 strains among three EV71 groups at 14 and 28 d post-inoculation. Thus, even though the vaccine strains were of the same genotype (C4a) and were administered at the equivalent dosage of 6.50 lg PFU/mouse, different levels of immunogenicity were observed.

### 2. NAbs induced by EV71 FCP

The anti-EV71 NAb seropositivity rates of groups immunized with the three FCPs ranged from 96.7–100% at 7 d, and were higher than 83.3% from 7 d to 84 d. The seroconversion rates induced by B2 and B3 (93.3–100%) were higher than that of B1 (83.3–100%) from 7 to 84 d post-inoculation with a dose of 500 U/mouse; the differences were not significant except at 14 d post-inoculation (*P*<0.01, Table 3).

**Table 3:** NAb seropositivity rates (dilution ≥1∶8) of mice inoculated with EV71 FCPs and FCP-As from three different manufacturers.

Sample	Manufacturer(Code)	Seropositivity rate (%)					
		7 d	14 d	21 d	28 d	56 d	84 d
FCPs (500 U/mouse)	A(B1)	100.0 (30/30)	83.3* (25/30)	93.3 (28/30)	86.7 (26/30)	95.0 (19/20)	95.0 (19/20)
	B(B2)	96.7 (29/30)	100.0 (30/30)	100.0 (30/30)	93.3 (28/30)	100.0 (20/20)	100.0 (20/20)
	C(B3)	100.0 (30/30)	100.0 (30/30)	96.7 (29/30)	100.0 (30/30)	100.0 (20/20)	100.0 (20/20)
FCP-As (200 U/mouse)	A(V1)	100.0 (20/20)	100.0 (20/20)	100.0 (20/20)	95.0 (19/20)	100.0 (20/20)	100.0 (20/20)
	B(V2)	100.0 (20/20)	100.0 (20/20)	95.0 (19/20)	100.0 (20/20)	100.0 (20/20)	100.0 (20/20)
	C(V3)	100.0 (20/20)	100.0 (20/20)	100.0 (20/20)	100.0 (20/20)	100.0 (20/20)	95.0 (19/20)

Immunization and NAb detection were carried out in three collaborative labs according to the same SOP. Twenty to thirty mice were immunized in each dose group. Results were highly repeatable in the three labs and were combined for statistical analysis.

* Anti-EV71 seropositive rate induced by B1 was significantly lower than that of B2 and B3 at 14 d post-inoculation, χ^2^ = 10.61, *P<0.01*.

There were differences in NAb GMTs and dynamic changes induced by the three FCPs (Table 4). Over time after immunization, the NAb GMTs of B2 slowly rose from 118.0 at 7 d to 259.9 at 84 d, while those of B3 gradually reduced (from 138.3 at 7 d to 23.5 at 84 d), and those of B1 remained stable (from 37.4 at 7 d to 26.5 at 84 d) with the lowest titers. The NAb GMTs of B1 were significantly lower than those of B2 and B3 from 7–21 d; however, the NAb GMTs of B2 were significantly higher than those of B1 and B3 from 21–84 d (Table 4). On the whole, the relative order of the three FCPs by NAb GMT was B2>B3>B1, which was similar to the NAb responses to the EV71 strains reported above.

**Table 4:** Dynamic trend of neutralizing antibody GMTs (95%CI) for EV71 FCPs.

	Sample (Manufacturer)	Time post-immunization (d)					
		7	14	21	28	56	84
GMTs (95% CI)	B1 (A)	37.4	24.6	25.7	36.4	35.2	26.5
		(27.8–50.4)	(17.2–35.2)	(18.9–35.0)	(24.5–50.6)	(24.5–50.6)	(17.5–40.3)
	B2 (B)	118.0	100.5	135.2	129.1	182.7	259.9*
		(91.2–152.8)	(78.4–128.9)	(98.4–185.8)	(95.2–175.2)	(102.7–324.8)	(137.9–489.6)
	B3 (C)	138.3	117.5	51.9	48.2	55.9	23.5**
		(101.7–188.2)	(85.0–162.3)	(39.9–67.6)	(37.0–62.8)	(32.1–97.5)	(17.0–32.6)
***P value***	***B1 vs B2***	***<0.001***	***<0.001***	***<0.001***	***<0.001***	***<0.001***	***<0.001***
	***B1 vs B3***	***<0.001***	***<0.001***	***<0.05***	***0.2083***	***0.1878***	***0.7115***
	***B2 vs B3***	***0.4311***	***0.4535***	***<0.001***	***<0.001***	***<0.05***	***<0.001***

Female BALB/c mice (4–6 weeks old) were i.p. inoculated with three EV71 FCPs (B1, B2, and B3) containing 500 U antigen. The sera were isolated and tested at 7, 14, 21, 28, 56 and 84 d post-inoculation. NAb titers equal to and above 1∶1536 were assigned a value of 1∶1536. Common logarithmic transformation of the NAb titer raw data was used to calculate the GMT and CV. SPSS10.0 software was used for statistical analyses.

* Denotes a significantly higher NAb GMT for B2 at 84 d post-inoculation than that of B2 at 7 d and 14 d post-inoculation *(P<0.05)*.

** Denotes a significantly lower NAb GMT for B3 at 84 d post-inoculation than that of B3 at 7 d,14 d, 21 d, 28 d and 56 d post-inoculation *(P<0.05)*.

### 3. NAb induced by EV1 FCP-A

Unlike the EV71 FCPs, the NAb seroconversion rates of groups inoculated with one of the three EV71 FCP-As (200 U/mouse) ranged from 95.0–100.0% at each time point after immunization, and no significant differences in seropositivity were observed among the three FCP-As (Table 3), while the NAb GMTs were different (Table 5). The NAb GMTs induced by V1 (143.0, 7 d; 91.1, 28 d; and 259.9, 84 d) and V3 (127.8, 7 d; 57.6, 21 d; and 241.1, 84 d) were low with similar titers at 21 d or 28 d, but they were high at 7 d and 84 d. The highest NAb GMTs appeared at 84 d, with 2.9-fold (V1) and 4.2-fold (V3) increases compared with the GMTs at 21 d and 28 d, respectively. Meanwhile, the NAb GMTs elicited by V2 increased gradually (70.6, 7 d; 206.9, 28 d; and 456.3, 84 d) and reached the highest titer at 84 d, which was 6.5-fold higher than that observed at 7 d.

**Table 5:** Dynamic trend of neutralizing antibody GMTs (95%CI) for EV71 FCP-As.

	Sample (Manufacturer)	Time post-immunization (d)					
		7	14	21	28	56	84
GMTs (95%CI)	V1 (A)	143.0	95.7	93.2	91.1	197.7	259.9*
		(104.0–196.5)	(69.5–131.7)	(64.30–135.1)	(58.1–142.9)	(138.1–283.0)	(157.1–429.9)
	V2 (B)	70.6	123.7	80.4	206.9	298.5	456.3**
		(46.6–107.2)	(81.9–186.9)	(50.9–127.2)	(141.0–303.5)	(163.1–546.3)	(286.4–727.1)
	V3 (C)	127.8	108.3	57.6	74.6	92.7	241.1***
		(95.8–170.3)	(70.8–165.7)	(37.6–88.2)	(46.6–119.6)	(56.6–151.7)	(119.1–487.9)
***P value***	***V1 vs V2***	***<0.001***	***0.1420***	***0.4305***	***<0.001***	***<0.05***	***0.1403***
	***V1 vs V3***	***0.4676***	***0.4756***	***<0.05***	***0.3066***	***<0.001***	***0.8445***
	***V2 vs V3***	***<0.001***	***0.4461***	***<0.05***	***<0.001***	***<0.001***	***0.0998***

Female BALB/c mice (4–6 weeks old) were i.p. inoculated with three EV71 FCP-As (V1, V2, and V3) containing 200 U antigen. The sera were isolated and tested at 7, 14, 21, 28, 56 and 84 d post-inoculation. NAb titers equal to and above 1∶1536 were assigned a value of 1∶1536. Common logarithmic transformation of the NAb titer raw data was used to calculate the GMT and CV. SPSS10.0 software was used for statistical analyses.

* Denotes a significantly higher NAb GMT for V1 at 84 d post- inoculation than that of V1 at 14 d, 21 d and 28 d post-inoculation *(P<0.05)*.

** Denotes a significantly higher NAb GMT for V2 at 84 d post- inoculation than that of V2 at 7 d, 14 d and 21 d post-inoculation *(P<0.05)*.

*** Denotes a significantly higher NAb GMT for V3 at 84 d post- inoculation than that of V3 at 21 d, 28 d and 56 d post-inoculation *(P<0.05)*.

In comparing the three FCP-As at 200 U/mouse, similar NAb seroconversion rates were found (with slight variations) and the same trend of GMTs over time was observed (Table 3 and 5). The GMTs of the mice inoculated with the three FCP-As had the smallest differences (95.7, 123.7, and 108.3 with 1.3-fold difference) at 14 d, and the largest differences (197.7, 298.5, and 92.7 with 3.2-fold difference) at 56 d. The NAb GMT induced by V2 was significantly lower than that of V1 and V3 at 7 d, but significantly higher than that of V1 and V3 at 28 d and 56 d (*P*<0.05).

### 4. ED_50_ of EV1 FCP-A at 7 d after inoculation

In addition to studying the dynamic NAb response, the NAb response to four serial dilutions of FCP-As was studied at 7 d after inoculation. Similar to the NAb response at 7 d after inoculation reported above, 100% seroconversion rate was observed in the 200 U dose groups (Table 6), and the lowest NAb GMT was induced by V2 at 7 d after inoculation (NAb GMTs induced by V1, V2, and V3 were 106.3, 37.4, and 52.0, respectively).

**Table 6:** Seroconversion rates and ED_50_ values of mice inoculated with EV71 FCP-As from three different manufacturers.

Sample	Seroconversion rate at 7 d pos-inoculation (%)					ED_50_ (U)
	200 (U)	50 (U)	12.5 (U)*	3.1 (U)*	0.8 (U)*	
V1	100	86.7	80.0	30.0	3.3	6.1
V2	100	76.7	16.7	6.7	0.0	25.3
V3	100	93.3	83.3	53.3	33.3	2.5

Immunization and NAb detection were carried out in three collaborative labs according to the same SOP. Thirty mice were immunized in each dose group. Results were highly repeatable in the three labs and were combined for statistical analysis. ED_50_ was calculated using the Spearman-Karber method [34].

* There were significant differences in seropositive rates induced by the three vaccines at doses of 12.5 U, 3.1 U and 0.8 U (χ^2^ = 35.22, 15.51 and 18.86, *P*<0.01).

Every final product showed a good dose-response relationship. In response to a reduction in vaccine dose, the NAb-positive rate of the V3 group showed the smallest decrease among the three vaccines and V3 had the lowest ED_50_ value (Table 6). The positive rate of V3 group was still 33.3% when the dose was dropped to 0.8 U/mouse, while that of V1 group and V2 group were 3.3% and 0%, respectively. The NAb-positive rates and GMTs of the V2-inoculated mice all decreased faster than those immunized with V1 and V3, with significant differences in seropositive rates observed among three vaccines at doses of 12.5 U, 3.1 U, and 0.8 U (*P*<0.01). The ED_50_ values of V1, V2, and V3 were 6.1 U, 25.3 U, and 2.5 U, respectively. Based on seroconversion rates at 7 d post-inoculation, V2 had the highest ED_50_ value; however, this vaccine also induced the highest NAb GMTs from 21 d to 84 d post-inoculation. These results indicated that the NAb response of any one dose will not fully reflect the immunogenicity of the EV71 FCP-A at any time point post-inoculation.

### 5. Protective efficacy of EV71 FCP-As in suckling mice

As shown in Figure 2a, in the protective efficacy evaluation by maternal antibodies, V1, V2, and V3 at doses of 162, 54, and 18 U/0.5 ml/dam were used to immunize adult ICR female mice. EV71/048M3C2 was administered intracerebrally to the newborn suckling mice at 20 LD_50_. At 2–3 days after inoculation, suckling mice born to negative control dams began to show symptoms of EV71 infection, including slow movement and limb paralysis, eventually leading to death (Figures 2b, c, d and Figure S2). In the negative control and CA16 control groups, all suckling mice died 5 days after inoculation (of all two independent experiments, only one pup in the negative control group survived, yielding a survival rate of 3.7%). In comparison, only a few suckling mice died in the EV71 experimental groups. When dams were immunized with a dose of 162 U/mouse, the pup survival rates were 96.2–97.0% in all three FCP-A groups. When dams were immunized with a dose of 54 U/mouse, the pup survival rate in the V1 group was 0%, but those in the V2 and V3 groups were as high as 93.9–95.8%. At 18 U/dam, 92.0% of the suckling mice survived in the V3 group. The ED_50_ of V1, V2, and V3 were calculated to be 96.9, 34.8, and 12.3 U, a 2.79- to 7.89-fold difference in dose. These results indicate that V3 has better protective effects against the 048M3C2 virus strain than V1 or V2.

## Discussion

Immunogenicity and protective efficacy in animal models are two key indexes required for the approval of a new vaccine [35], [36]. Several animal models have been developed for testing EV71 vaccines [16]–[18], [20], [22], [35], including the maternal–NAb mouse model, which has been used to evaluate the protective efficacy of these vaccines [16], [18], [20]. However, the lack of standard methods for measuring EV71 antigen content and NAb titer have been major limitations in the evaluation and comparison of vaccine immunogenicity [37], [38]. Two recent reports from Chinese Taiwan and mainland China addressed this problem by establishing assays to measure antigen content using a VP2 monoclonal antibody (MAb) and a VP1 MAb with high neutralizing activity [29], [39]. Prior to these recent studies, the antigenicity of EV71 bulks from three manufacturers was compared with the EV71 antigen standard by kits using the VP1 MAb. The EV71 antigen reference standard demonstrated good parallelism and linearity with the different vaccine antigens, and the R^2^ values were all higher than 0.999 [30]. These findings suggested that the vaccine antigens produced by the three manufacturers could be accurately quantitated using the same units of measure (U/ml) when using this kit and standard. In addition, the CPE assays performed in each collaborative lab followed the same SOP and employed the national reference standards for neutralizing antibody as the quality control. Only when the results of three reference standards and virus back-titration all met the detection range allowed, were the data of CPE assays accepted. To further confirm the results of CPE assays, when all CPE assays were completed, 120 samples were randomly selected and tested again; the differences among all 120 samples between two tests were within 4-fold of each other, indicating that the CPE assays have good repeatability. These data suggest that the use of a standard *in vitro* neutralization test is critical to confirm the repeatability, comparability, and accuracy of NAb titer measurements, which are essential for the proper comparison of the immunogenicity and protective effects of vaccines from different manufacturers.

The immunogenicity of antigens in inactivated virus vaccines is crucial for successful vaccine development [15], [26]. We previously compared the genomes of 11 EV71 vaccine strains in China that have differences in nucleotide and amino acids in VP1–VP3 regions [26]. To compare the immunogenicity of the vaccines in this study, the three vaccine strains were diluted to an equivalent virus titer (6.50 lg PFU/mouse) before immunization. Although they are all of the C4a genotype,different NAb GMTs were induced by the vaccine strains, which was similar to the results obtained by Wang et al. and Chang et al. [40], [41]. In the study by Liu et al [42], two peptides (211–220aa in VP1 and 136–150aa in VP2) were considered to be potentially of greater importance for influencing NAb responses. In our studies, only one residue of these peptides was different (M1, M2, M3 is T, S, T at 144aa in VP2) among the three vaccine strains. Whether this differential residue has an effect on the immunogenicity of the vaccine strains is an important area of future investigation. Chang et al. reported that formaldehyde inactivation may influence the immunogenicity of the C4 strain [41]. However, our research showed that the EV71 immunogenicity of all three vaccine strains to induce NAbs did not decrease after inactivation, indicating that the key conformational epitopes were not affected by formaldehyde treatment.

The seroconversion rate induced by FCPs (500 U) and FCP-As (200 U) were higher than 80% and 95%, respectively, and the NAb titers induced by FCP-As (200 U) were all higher than those induced by FCPs (500 U). The seroconversion rates and the NAb titers produced by vaccines from the three manufacturers were relatively close. These results suggest that the aluminum hydroxide adjuvant used at similar concentrations (1.00–1.16 mg/ml) in the EV71 vaccines from different manufacturers had different immunological enhancing effects (Figure S3). Combined with the comparisons in a recent report [41] of an EV71 vaccine absorbed with either aluminium phosphate adjuvant or aluminium hydroxide adjuvant, we can conclude from our results that different types of alum adjuvant and different manufacturing processes may influence the degree of immunogenicity enhancement [43], [44]. Compared with the FCPs, the differences in immunogenicity among the FCP-As produced by the three manufacturers were reduced, especially at 14 d and 21 d after immunization. These results confirmed that the levels of immunogenicity of FCP-A produced by the three manufacturers were similar, and may provide the basis for establishing a uniform antigen unit (U) for EV71 vaccine dosages in clinical trials.

However, the ED_50_ for the NAb response and the *in vivo* protection of the three vaccines were different. The V2 vaccine elicited the most potent NAb response at 28–84 d after inoculation; however, its ED_50_ with regards to both *in vivo* protection and NAb response were higher than V3. There are several possible reasons for this observation: 1) the efficacy of the V2 vaccine may be most affected by dilution, as its protein content was the lowest among the three vaccines; 2) differences in the cell substrates and processes used during production may lead to different ratio of E-type particles (empty) and F-type particles (full) in the vaccines;3) the effects of different aluminium adjuvant adsorption technologies used by the three different manufacturers may contribute to the range of antibody responses. This result demonstrated that evaluating NAb responses at a single time point or using a single index is not adequate for evaluating the immunogenicity of the EV71 vaccines. However, the index of ED_50_ was suitable for monitoring the vaccine production from different manufacturers.

The pup EV71 challenge model for evaluating protective efficacy by maternally transferred antibodies was applied in this study as an indirect method for comparing the protective efficacy of the inactivated virus vaccines [16], [18], [20]. In an earlier study carried out by Bek et al., all mice immunized with more than 50 U of vaccine antigen could survive when challenged with a mouse adapted strain (B3 genotype) of EV71 [27]. Additionally, in a report by Dong et al., another inactivated vaccine (with dose of 40 EU or 160 EU) exhibited nearly 100% immunoprotective efficacy in mice and monkeys that were challenged by the FY-23 virus (C4 genotype of EV71) [28]. However, the immunoprotective effects of the vaccines from different manufacturers are not comparable because of differences in animal experimental design and challenge virus.

By titrating the vaccines in this study, we found that every final product showed a good dose-response relationship. After only one immunization at a dose of 162 U/0.5 ml/dam, the protection rate passed 90%, consistent with the NAb seropositivity rate and GMTs of the three final products at 21 d post-inoculation. In particular, V3, produced by manufacturer C, showed good protective effects at a dose of only 18 U/0.5 ml/dam, which was better than that observed with V1 or V2. These results were consistent with the NAb responses in the vaccine titration experiment, in which the NAb-positive rate elicited by V3 showed the best ED_50_ among the three vaccines. These results were also consistent with a study reported by Bek et al. [27], which showed that the higher protection level conferred by the inactivated virus vaccine mainly correlated with the NAb titer elicited, although the ED_50_ value for protecting suckling mice was not absolutely related with ED_50_ value for NAb. However, some researchers have speculated that *in vitro* assays do not provide a full representation of the *in vivo* activity and functions of pathogen-specific immunoglobulin, as *in vivo* IgG can activate complement cascades, participate in opsonization, and mediate antibody-dependent cytotoxicity, all of which play an important role in the protective process [22].

EV71 has only one serotype but 11 subtypes including A, B (B1–B5), and C (C1–C5). C4 has been the predominant subtype in mainland China in recent years, and therefore three C4 genotype strains were selected as vaccine strains. Whether these EV71 vaccines of C4 genotype can protect against the EV71 infection caused by other genotypes is another key indicator in the evaluation of vaccine immunogenicity. However, there are few studies addressing this matter. Among all subtypes, the genetic homology between the C4 subtype and A genotype is the lowest. In previous studies conducted in our lab, we investigated the cross-neutralizing reaction capacity between 10 strains of C4 subtype (including 3 vaccine strains) and one A genotype strain. The results indicated that all 10 strains of C4 subtype had a cross-neutralizing capacity towards the A genotype strain, but the magnitude varied [26]. Bek et al. reported that EV71 vaccine of the C4 subtype can effectively protect mice from the lethal challenge of B3 subtype EV71 [27]. However, the cross-protective capacity of these EV71 vaccines (C4 subtype) against other subtypes of EV71 needs further study.

In summary, using uniform antigen units (U/ml) and a standardized NAb assay, protective effects and NAb responses induced by three EV71 inactivated vaccines were studied at the vaccine strain (activated and inactivated) stage as well as the final product stage without adjuvant (FCP) or with adjuvant (FCP-A). Although the three products had differences in the sequences of the vaccine strains as well as manufacturing process, the three FCP-As (200 U) showed good immunogenicity, inducing NAbs at a rate of greater than 95% in mice at 7 d after one immunization, with the NAb titer gradually increasing thereafter. The antigen content of the three EV71 vaccines, which are being used in current clinical trials, ranged from 162 U/dose to 506 U/dose. Our analysis indicated that the lowest dose (162 U/dose) among the three FPC-As showed good protective effects, with 90–100% of suckling mice surviving lethal challenge. This study compared the immunogenicity of different inactivated EV71 vaccines, and provided a basis for evaluating immunogenicity during clinical trials, indicating that the comparison of vaccine effects at one time point may not be representative of other time points. Finally, the standardized EV71 antigen unit (U/ml) should be used to evaluate the antibody responses and protective efficacy of EV71 vaccines in future clinical trials.

## Supporting Information

Figure S1 **Diagrammatic drawing of comparative studies for NAbs induced in mice by EV71 FCPs and FCP-As from three different manufacturers.**
(TIF)

Figure S2 **Immune protection experiment–symptoms displayed by suckling mice.** (**a**) Healthy suckling mice. (**b**) Inoculated suckling mice with disease onset on 5th day (arrows: rear limb paralysis).
(TIF)

Figure S3 **Dynamic trend analysis of neutralizing antibody GMTs for EV71 FCPs and FCP-As from different manufacturers.** * Denotes significant differences between EV71 FCPs and FCP-As (*P<0.05*).
(TIF)

Table S1 **The targets and projects were researched in this paper.**
(DOC)
