# Supplementary figures and images for: Comparative Analysis of the Immunogenicity and Protective Effects of Inactivated EV71 Vaccines in Mice
Source: PLoS One. 2012 Sep 28;7(9):e46043. doi: 10.1371/journal.pone.0046043 (PMC3460965; doi:10.1371/journal.pone.0046043)

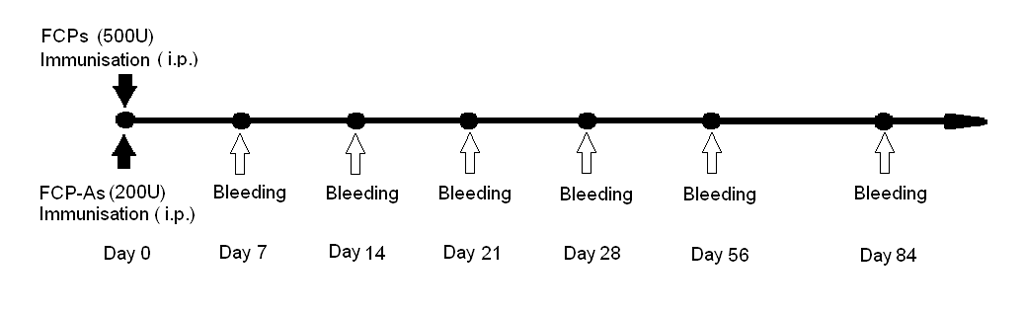

Supplement: Figure S1 — Diagrammatic drawing of comparative studies for NAbs induced in mice by EV71 FCPs and FCP-As from three different manufacturers. (TIF) [file pone.0046043.s001.tif]

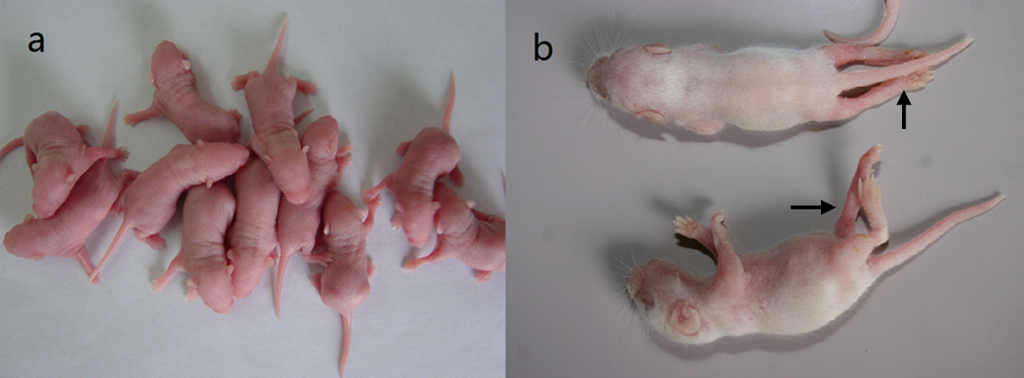

Supplement: Figure S2 — Immune protection experiment–symptoms displayed by suckling mice. (a) Healthy suckling mice. (b) Inoculated suckling mice with disease onset on 5th day (arrows: rear limb paralysis). (TIF) [file pone.0046043.s002.tif]

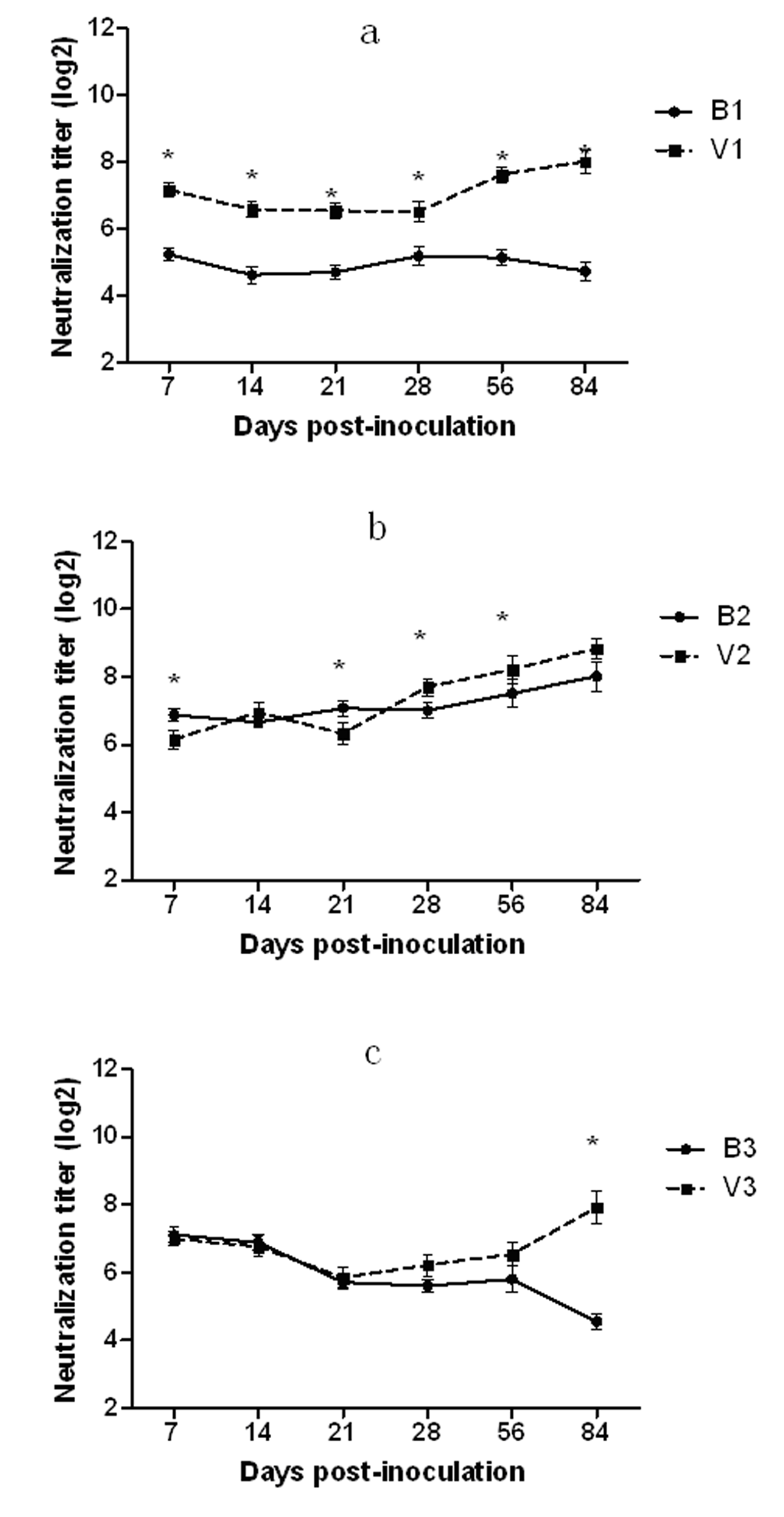

Supplement: Figure S3 — Dynamic trend analysis of neutralizing antibody GMTs for EV71 FCPs and FCP-As from different manufacturers. * Denotes significant differences between EV71 FCPs and FCP-As (P<0.05). (TIF) [file pone.0046043.s003.tif]
