# Supplementary material for: Comparative Analysis of the Immunogenicity and Protective Effects of Inactivated EV71 Vaccines in Mice
Source: PLoS One. 2012 Sep 28;7(9):e46043. doi: 10.1371/journal.pone.0046043 (PMC3460965; doi:10.1371/journal.pone.0046043)
Supplement: Table S1 — The targets and projects were researched in this paper. (DOC) [file pone.0046043.s004.doc]

**Table S1.** The targets and projects were researched in this paper

| **Sample** | **The research project** | | |
| --- | --- | --- | --- |
|  | **NTAb response** | **ED_50_ detection** | **Protection** |
| EV71 strains | **√** | **-** | **-** |
| Inactivated EV71 strains | **√** | **-** | **-** |
| FCPs | **√** | **-** | **-** |
| FCP-As | **√** | **√** | **√** |
